# Long non‐coding RNA 01126 promotes periodontitis pathogenesis of human periodontal ligament cells via miR‐518a‐5p/HIF‐1α/MAPK pathway

**DOI:** 10.1111/cpr.12957

**Published:** 2020-11-24

**Authors:** Mi Zhou, Hui Hu, Yineng Han, Jie Li, Yang Zhang, Song Tang, Yu Yuan, Xiaonan Zhang

**Affiliations:** ^1^ College of Stomatology Chongqing Medical University Chongqing China; ^2^ Chongqing Key Laboratory of Oral Diseases and Biomedical Sciences Chongqing China; ^3^ Chongqing Municipal Key Laboratory of Oral Biomedical Engineering of Higher Education Chongqing China; ^4^ Department of Oral and Maxillofacial Surgery Peking University School and Hospital of Stomatology Beijing China

**Keywords:** HIF‐1α, hypoxia, long non‐coding RNA, miR‐518a‐5p, periodontitis

## Abstract

**Background:**

Periodontitis is a prevalent oral inflammatory disease, which can cause periodontal ligament to a local hypoxia environment. However, the mechanism of hypoxia associated long non‐coding RNAs (lncRNAs) involved in periodontitis is still largely unknown.

**Methods:**

Microarray was performed to detect the expression patterns of lncRNAs in 3 pairs of gingival tissues from patients with periodontitis and healthy controls. The expression of lncRNA 01126 (LINC01126), miR‐518a‐5p and hypoxia‐inducible factor‐1α (HIF‐1α) in periodontal tissues and in human periodontal ligament cells (hPDLCs) under hypoxia was measured by quantitative real‐time polymerase chain reaction or western blot. Fluorescence in situ hybridization and cell fraction assay were performed to determine the subcellular localization of LINC01126 and miR‐518a‐5p. Overexpression or knockdown of LINC01126 or HIF‐1α was used to confirm their biological roles in hPDLCs. MTT assays were performed to evaluate hPDLCs proliferation ability. Flow cytometry was used to detect apoptosis. ELISA was used to measure the expression levels of interleukin (IL)‐1β, IL‐6, IL‐8 and TNF‐α. Dual‐luciferase reporter assays were performed to assess the binding of miR‐518a‐5p to LINC01126 and HIF‐1α. RNA immunoprecipitation assay was used to identify whether LINC01126 and miR‐518a‐5p were significantly enriched in AGO‐containing micro‐ribonucleoprotein complexes.

**Results:**

We selected LINC01126, which was the most highly expressed lncRNA, to further verify its functions in periodontitis‐induced hypoxia. The expression of LINC01126 was increased in periodontal tissues. *In vitro* experiment demonstrated that LINC01126 suppressed proliferation, promoted apoptosis and inflammation of hPDLCs under hypoxia via sponging miR‐518a‐5p. Moreover, we identified HIF‐1α acted as a direct target of miR‐518a‐5p in hPDLCs and LINC01126 promoted periodontitis pathogenesis by regulating the miR‐518a‐5p/HIF‐1α/MAPK pathway.

**Conclusion:**

LINC01126 promotes periodontitis pathogenesis of hPDLCs via miR‐518a‐5p/HIF‐1α/MAPK pathway, providing a possible clue for LINC01126*‐*based periodontal therapeutic approaches.

## Graphical abstract

LINC01126, which was the most highly expressed lncRNA, to further verify its functions in periodontitis‐induced hypoxia. The expression of LINC01126 were increased in periodontal tissues. In vitro experiment demonstrated that LINC01126 suppressed proliferation, promoted apoptosis and inflammation of hPDLCs under hypoxia via sponging miR‐518a‐5p. Moreover, we identified HIF‐1α acted as a direct target of miR‐518a‐5p in hPDCLs and LINC01126 promoted periodontitis pathogenesis by regulating the miR‐518a‐5p/HIF‐1α/MAPK pathway. In brief, LINC01126 promotes periodontitis pathogenesis of hPDLCs via miR‐518a‐5p/HIF‐1α/MAPK pathway, providing a possible clue for LINC01126‐based periodontal therapeutic approaches.

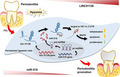

**Abbreviations**
BP: biological process
CC: cellular component
ceRNAs: competing endogenous RNAs
DMEM: Dulbecco’s modified Eagle’s medium
ELISA: enzyme‐linked immunosorbent assay
ERK: extracellular signal‐regulated kinase
FC: fold change
FISH: Fluorescence in situ hybridization
GAPDH: housekeeping gene glyceraldehyde‐3‐phosphate dehydrogenase
GO: gene ontology
GSEA: gene set enrichment analysis
HIF‑1: hypoxia‑inducible factor‑1
HIF‑1α: hypoxia‑inducible factor‑1
hPDLCs: human periodontal ligament cells
IL: interleukin
JNK: c‐jun N‐terminal kinase
lncRNA: long non‐coding RNA
MAPKs: mitogen‐activated protein kinases
MF: molecular function
miRNA: microRNA
ncRNAs: Non‐coding RNAs
PDL: periodontal ligament
PVDF: polyvinylidene difluoride
qRT‐PCR: quantitative real‐time polymerase chain reaction
RIP: RNA immunoprecipitation
SD: standard deviation
α‐MEM: α‐modified Eagle’s medium

## 1 INTRODUCTION

Periodontitis, malocclusion and caries are the three major public oral diseases worldwide, which is attracting increasing attention. Periodontitis is reported to have an over 50% of incidence among adults and approximately 5‐20% of adults suffer from severe periodontitis worldwide.^1^, ^2^, ^3^ The periodontal ligament (PDL), a soft tissue connecting the teeth and alveolar bone, plays an important role during periodontitis and orthodontics.^4^, ^5^ It is involved in several physiological functions like maintaining dental homeostasis, resisting inflammation and remodelling alveolar bone.^6^, ^7^ Periodontitis can severely damage the surrounding vasculature, promote subgingival microorganisms proliferation, further leads PDL to a low‐oxygen microenvironment.^8^ When chronic inflammation is present, human periodontal ligament cells (hPDLCs) are susceptible to hypoxia and can induce various molecular responses to adapt to the hypoxic condition.^9^, ^10^ hPDLCs are the dominant type of cell in the PDL, having multiple functions including producing principal fibres and dental cement, transportation, and mineralization synthesizing extracellular matrixs.^11^, ^12^, ^13^ These capacities can be inhibited by many factors including hypoxia though the underlying mechanism remain unknown.^14^, ^15^ Therefore, further exploring the regulation mechanism under hypoxia of hPDLCs would be helpful to clinical periodontal treatment.

Non‐coding RNAs (ncRNAs), a large group of RNAs without protein‐coding functions, are drawing attention in their important roles in regulating transcripts during biological and pathological process.^16^ Long non‐coding RNAs (lncRNAs), which is longer than 200 nucleotides in length, are an important subset of ncRNAs.^17^ Previous studies have revealed several lncRNAs and its associated competing endogenous RNAs are aberrantly expressed in periodontitis via bioinformatics analysis.^18^, ^19^ Periodontitis pathogenesis can cause the hPDLCs into a local hypoxic environment, however, only a few lncRNAs, including HIF1‐AS1 and HIF1‐AS2 have been investigated to explain their hypoxia‐induced functions in hPDLCs.^20^ A large amount of lncRNAs and the potential mechanisms are still unknown in this field. Functionally, a majority of lncRNA can bind to microRNA (miRNA) binding sites, forming a regulatory competing endogenous RNAs (ceRNAs) network (lncRNAs‐miRNAs‐mRNAs), and further regulate the targeted mRNAs of miRNAs.^21^, ^22^ MiRNAs can negatively regulate gene expression of mRNAs by directly binding to the 3′‐UTR region of mRNAs.^23^ These regulatory networks are involved in multiple cellular processes.^24^, ^25^

Long intergenic non–protein‐coding RNA 1126 (LINC01126, NR_027251.1), a recently discovered lncRNA is located on chromosome 2 (2p21) in humans. It was first reported in 2002 containing 1645 bp.^26^ However, the function and the regulatory mechanism of LINC01126 in hPDLCs under hypoxia have never been elaborated. Therefore, in present study, we investigated the role of LINC01126 in proliferation capacity, apoptosis and inflammation of hPDLCs under hypoxia and further elucidate how lnc01126 regulates these process via LINC01126/miR‐518a‐5p/HIF‐1α/MAPK pathway. These findings will better elucidated how hypoxia affects proliferation, apoptosis and inflammation via LINC01126 in hPDLCs, making us better understand the aetiology and therapy of periodontitis treatment.

## 2 MATERIALS AND METHODS

### 2.1 Cell culture

Human embryonic kidney (293T) cells were purchased from the American Type Culture Collection (Manassas, VA, USA). hPDLCs were isolated and cultured according to previous published protocols.^27^ Teeth were obtained from premolar teeth extracted from healthy patients (mean 13 years old) for orthodontic treatment. Informed consent was given to all the patients involved, and the research protocol had been approved by the Human Ethics Committees of Chongqing Medical University. 293T cells were routinely cultured in Dulbecco’s modified Eagle’s medium (DMEM, Gibco, Grand Island, NY, USA), and hPDLCs were routinely cultured in α‐modified Eagle’s medium (α‐MEM, Gibco) at 37℃ with 5% CO_2_. Both of the cells were supplemented with 10% foetal bovine serum (Gibco) and 1% penicillin/streptomycin (Gibco). To investigate the role of hypoxia on hPDLCs, hPDLCs were divided into 3 groups randomly as: 1% O_2_ content, severe hypoxia group, 5% O_2_ content, slight hypoxia group and 21% O_2_ content, the control group. Cells in the two Hypoxic conditions were cultured in a 3‐gas (N_2_/O_2_/ CO_2_) incubator (NuAire Inc., Plymouth, MN), while the normoxia group was cultivated in a normal CO_2_ incubator (NuAire, Inc.).

### 2.2 Gingival Biopsies collection

Human gingival tissues were obtained from patients with periodontitis (n = 47; mean age, 31 ± 1.8 years) or from healthy controls who received gingivectomy or crown lengthening during orthodontic or prosthodontic treatment (n = 19; mean age, 28.3 ± 2.4 years). All protocols dealing with patients were approved by the Ethics Committee of Stomatological Hospital of Chongqing Medical University, Chongqing, People’s Republic of China (2019 NO.88) and all the patients signed the Informed consent. After collection, periodontitis biopsies were submerged in TRIzol reagent for RNA extraction and further quantitative real‐time polymerase chain reaction (qRT‐PCR) measurement to determine the expression of lncRNAs, miRNA and HIF‐1α (primers listed in Table S1). Details were illustrated in the RNA extraction section.

### 2.3 Microarray analysis

Total RNA was extracted from 3 paired human gingival tissues from patients with periodontitis and healthy controls. Tissue samples preparation and microarray hybridization were performed according to the manufacturer's standard protocols. The acquired raw data and array images were extracted with Agilent Feature Extraction (version11, Agilent, USA) software. GeneSpring GX v12.0 (Agilent) was used for quantile normalization and subsequent data processing. The microarray was provided by KangChen Bio‐tech (Shanghai, China). We identified aberrantly expressed lncRNAs as those with statistical significance (*P* < .05; fold change (FC) ≥ 1.5). Gene set enrichment analysis (GSEA) was performed using GSEA software (v. 3.0) and illustrated by ClusterProfile. Gene Ontology (GO) classification including GO‐BP (biological process), GO‐MF (molecular function) and GO‐CC (cellular component) were visualized by the DAVID V6.8 (http://david.ncifcrf.gov) webserver.

### 2.4 RNA Immunoprecipitation (RIP) Assay

RIP assay was performed using the EZ‐Magna RIP Kit (Merck Millipore, Darmstadt, Germany) according to the manufacturer’s instruction. Firstly, magnetic beads were incubated with AGO2 antibody (Cell Signaling Technology, Beverly,MA) and the negative control immunoglobulin G (IgG) (Merck Millipore), respectively. Then, the cells were lysed in RIP Lysis buffer and the lysate was incubated with different bead‐antibody complexes for 1 night at 4 ℃. The next day, to digest the protein and isolate the immunoprecipitated RNA, samples were incubated with Proteinase K by shaking. After that, qRT‐PCR was carried out to analyse and the results were presented as fold enrichment in AGO2 relative to input.

### 2.5 Fluorescence in situ hybridization (FISH)

FISH assay was performed using the Fluorescent in situ Hybridization Kit (RiboBio, Guangzhou, China) following the manufacturer’s instructions. Briefly, cells were rinsed in PBS and fixed in 4% formaldehyde for 15 min at room temperature. Then, the cells were permeabilized in PBS containing 0.5% Triton X‐100 at 4℃ for 5 min and washed with PBS three times for 5 minutes each time. After that, the sample was pre‐hybridized at 55 ℃ for 30 min. For hybridization, anti‐LINC01126, anti‐miR‐518a‐5p probes were added in the hybridization solution and cells were incubated at 37 ℃ overnight in the dark. The next day, the cells were counterstained with DAPI and images were taken using a confocal laser‐scanning microscope (Carl Zeiss, Oberkochen, Germany).

### 2.6 MTT assay

hPDLCs were seeded into 96‐well plates (1x10^4^ cells/well). After various treatment, 500 lg/mL of [3–(4, 5‐dimethylthiazol‐2‐yl)‐2,5‐ diphenyltetrazolium bromide solution (MTT; Sigma‐Aldrich, St. Louis, MO, USA) was added to each well at indicated time points and then incubated for 4 h at 37℃. After that, dimethyl sulphoxide solution (200 lL, Sigma‐Aldrich) was added in each well to melt the reaction product formazan. Optical density was measured at 490 nm using a micro‐plate reader (BioRad Model 550; Hercules, CA, USA).

### 2.7 RNA oligoribonucleotides and vectors construction

The RNA oligoribonucleotides include miR‐518a‐5p mimic, miR‐518a‐5p inhibitor and the corresponding miRNA control (mimic‐NC, inhibitor‐NC) were purchased from Integrated Biotech Solutions Co. (Ibsbio Co., Shanghai, China; Table S2). Recombinant lentiviruses containing full‐length LINC01126, HIF‐1α and the scramble control (NC) were obtained from Ibsbio Co.. Recombinant lentiviruses targeting LINC01126 (sh‐01126) and the scramble control (sh‐NC) were obtained from Ibsbio Co. (Shanghai, China). The pCDH vectors schematic diagram of pCDH vectors was shown in Figure S2. Wide‐type (LINC01126‐WT, HIF‐1α‐WT) or mutant type (LINC01126‐MUT, HIF‐1α‐MUT) containing miR‐518a‐5p binding sites were constructed and cloned into the downstream of the luciferase gene in luciferase vectors (Ibsbio Co.).

### 2.8 Cell transfection

For Transient transfection, cells were first seeded in 6‐well plates. When cell confluence reached 80%, 100 nM miRNA inhibitor or mimic or siRNAs or 2 μg plasmid were transfected into cells using Lipofectamine 3000 (Invitrogen, Carlsbad, CA, USA) following the manufacturer’s instruction. For stable transfection, hPDLCs were exposed to dilutions of the viral supernatants for 72 h. After that, puromycin was added to select the stably transfected cells.

### 2.9 Dual‐luciferase reporter assay

Cells were seeded in 48‐well plates, and a mixture of 40 ng luciferase reporter, 4 ng Renilla, and 100 nM miR‐518a‐5p mimic or control was transfected into cells with Lipofectamine 3000 (Invitrogen). After 24 h, activity of luciferase was determined using the Dual‐Luciferase Reporter Assay System (Promega, Beijing, China) normalized to Renilla luciferase.

### 2.10 Apoptosis assay

After various treatment, hPDLCs were harvested and washed in PBS. Then, the cells were resuspended in fixation fluid. FITC Annexin V (50 lg/mL RNase A) (Sigma‐Aldrich) was added to the cell suspension for incubation at room temperature in dark. After 1 h, cell apoptosis was assessed by the FACScan system (BioRad).

### 2.11 Total RNA extraction and qRT‐PCR

Total RNA was extracted from cultured cells using TRIzol reagent (Invitrogen) according to the manufacturer’s instructions. A cDNA Reverse Transcription kit (Takara, Tokyo, Japan) was used to synthesize cDNA from 2 µg of total RNA. The SYBR Green Master Mix (Roche, Basel, Switzerland) was used for qRT‐PCR on an ABI 7500 cycler (Applied Biosystems, Foster City, CA, USA). Thermal settings were as follows: 95°C for 10 min, 40 cycles of 95°C for 15 s and 60°C for 1 min. The mean of the housekeeping gene β‐actin was acted as an internal reference for mRNA and small nuclear RNA U6 as the internal control for micro miRNA. qRT‐PCR was performed three times. The primers used in the experiment are listed in Table S3, and the results were analysed using the 2^−ΔΔCt^ relative expression method.

### 2.12 Enzyme‐linked immunosorbent assay (ELISA)

The culture supernatant of hPDLCs was collected from the 6‐well plates, and the inflammatory cytokine interleukin (IL)‐1β, IL‐6, IL‐8 and TNF‐α was quantified using human ELISA kits (Proteintech Group, Rosemont, IL, USA) following the manufacturer’s protocols, and normalized to cell protein concentrations at an absorbance of 405 nm.

### 2.13 Cell fractionation assays

A Nuclei Isolation kit (Invent Biotechnologies, Beijing, China) was used for the cell fractionation assay to obtain cytoplasmic and nuclear RNA according to the manufacturer’s protocols. Briefly, cells were harvested after treatment and appropriate amounts of cytoplasm extraction buffer were added to cell pellets. Cells were then vortexed vigorously for 15 s and the mixtures were incubated on ice for 5 min. Subsequently, cells were centrifuged at top speed in a microcentrifuge at 4°C for 5 min; the supernatant (cytosol fraction) was then transferred to a pre‐chilled 1.5‐ml tube, and the remaining pellet was used as the nuclear fraction. Then, TRIzol (Invitrogen) was used to extract RNA from each fraction as described above.

### 2.14 Western blot

Total protein from cultured cells was extracted using radio immunoprecipitation assay lysis buffer. 8 % sodium dodecyl sulphate–polyacrylamide gel electrophoresis was used to separate equal quantities of proteins. Then, the proteins were transferred onto polyvinylidene difluoride (PVDF) membranes (Merck Millipore). After incubating in 5% milk‐TBST for 1h, the membranes were incubated at 4°C overnight in primary antibodies against HIF‐1α (1:1000; Abcam, Cambridge, UK) and β‐actin (1:1,000; Abcam). The next day, after the PVDF membranes were washed with TBST for three times the corresponding secondary antibodies (1:10,000; ZSGB‐BIO, Beijing, China) were used to incubate the membranes for 1 h. After washing three times with TBST, the ImageJ software (NIH, Bethesda, MD, USA) was used to quantify the intensities of the bands.

### 2.15 Statistical analysis

The statistical data were processed by SPSS 19.0 software (SPSS Inc., Chicago, IL, USA). One‐way ANOVA or Student’s t test was used to compare differences in groups. The data were presented as mean values ± standard deviation (SD). *P* < 0.05 was considered as statistically significant. Each experiment was repeated at least three times.

## 3 RESULTS

### 3.1 Aberrant expression of lncRNAs in periodontitis

**Figure 1:**
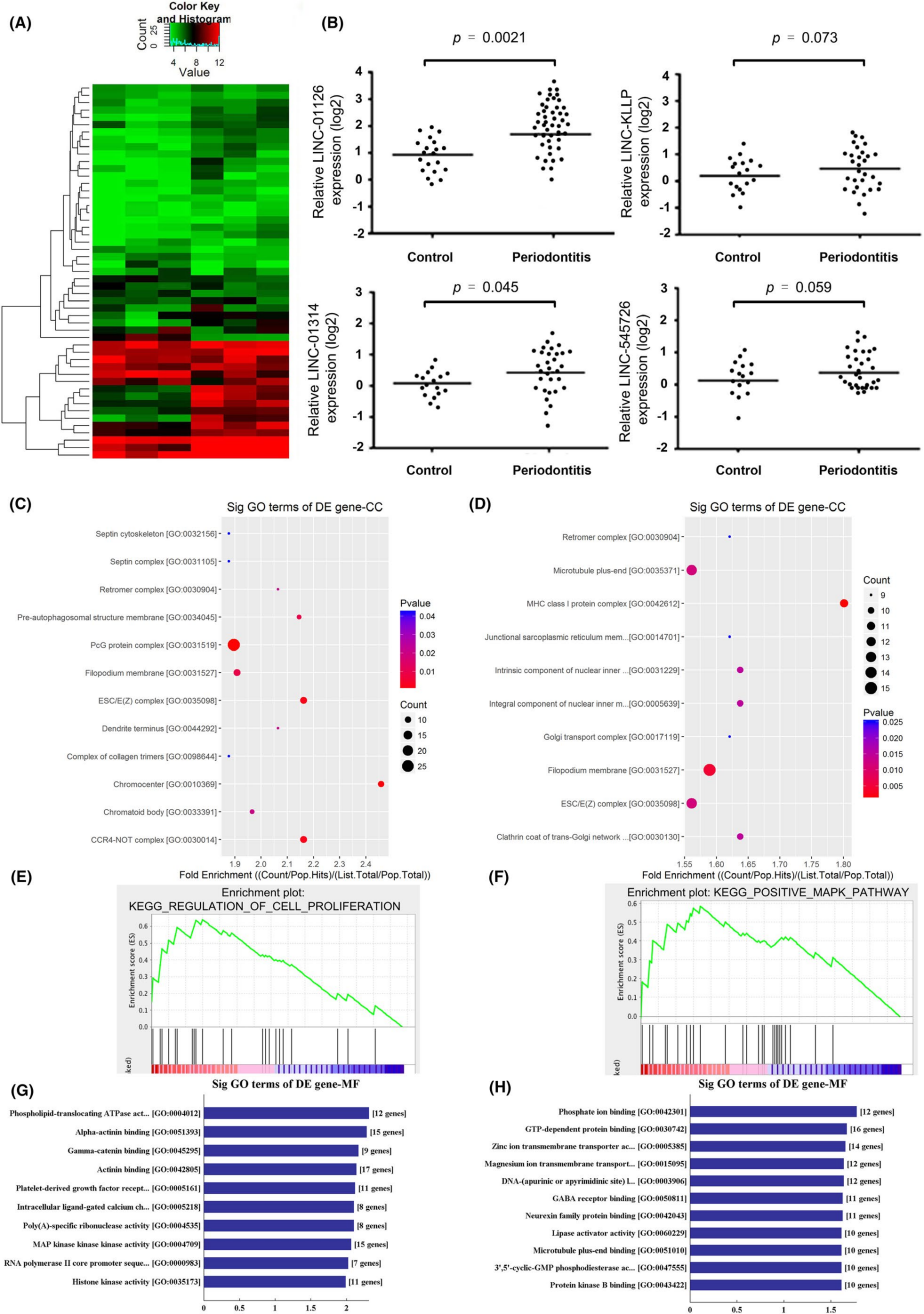
Aberrant expression of lncRNAs in periodontitis. (A) Heatmap of the top 50 differentially expressed lncRNAs (|log_2_FC| >1.5, *P* < .05) in 3 pairs of gingival tissues from patients with periodontitis and the healthy controls though lncRNA expression microarray. (B) RNA expressions of the 4 mostly upregulated lncRNAs LINC01126, LINC545726, lncRNA‐KLLP, LINC01314 in periodontal tissues and the controls group (C, D) The top 10 upregulated and downregulated GO functions of the cellular component (CC) domains. (E) GSEA plot of differentially expressed involved in proliferation in periodontal tissues. (F) GSEA plot of genes involved in MAPK signalling pathway in periodontal tissues. Running enrichment score was positive for most differentially expressed genes. (G, H) The top 10 upregulated and downregulated GO functions of the molecular function (MF) domains

We obtained the aberrantly expressed lncRNAs by analysing the microarray data. From 3 paired samples, we found that there were a total of 1080 lncRNAs significantly differentially expressed with fold changes greater than 1.5 and p value less 0.05 (|log_2_ FC| >1.5, p < 0.05). Among them, 589 lncRNAs were upregulated while 491 were downregulated in the periodontitis group (Figure 1A). The 4 mostly upregulated lncRNAs were LINC01126 (log_2_ FC = 6.31), LINC01314 (log_2_ FC = 6.27), lncRNA‐KLLP (log_2_ FC = 5.43) and LINC545726 (log_2_ FC = 6.96) (Figure 1B). Next, we compared the expression of these four candidate lncRNAs between gingival samples from patients with periodontitis (n = 47) and healthy controls (n = 19). The results showed that LINC001126 was significantly increased in periodontal groups (Figure 1B). GO and GSEA results indicated that these differentially expressed lncRNA were involved in the regulation of multiple inflammatory pathways, controlling proliferation and apoptosis (GO‐MF and GO‐CC) (Figure 1C‐H). GSEA plot demonstrated that the majority of differentially expressed genes were involved in the MAPK signalling pathway as the running enrichment score was positive for most of them (Figure 1F). The full‐length sequence, RNA secondary structure and location information of LINC 01126 are shown in Figure S1A‐C. Taken together, combined with the microarray results and the aberrant expression in periodontal tissues, we hypothesized that LINC01126 was highly correlated with periodontitis pathogenesis and we selected it to further verify its functions in periodontitis pathogenesis.

### 3.2 LINC01126 is upregulated in hypoxia‐induced hPDLCs

**Figure 2:**
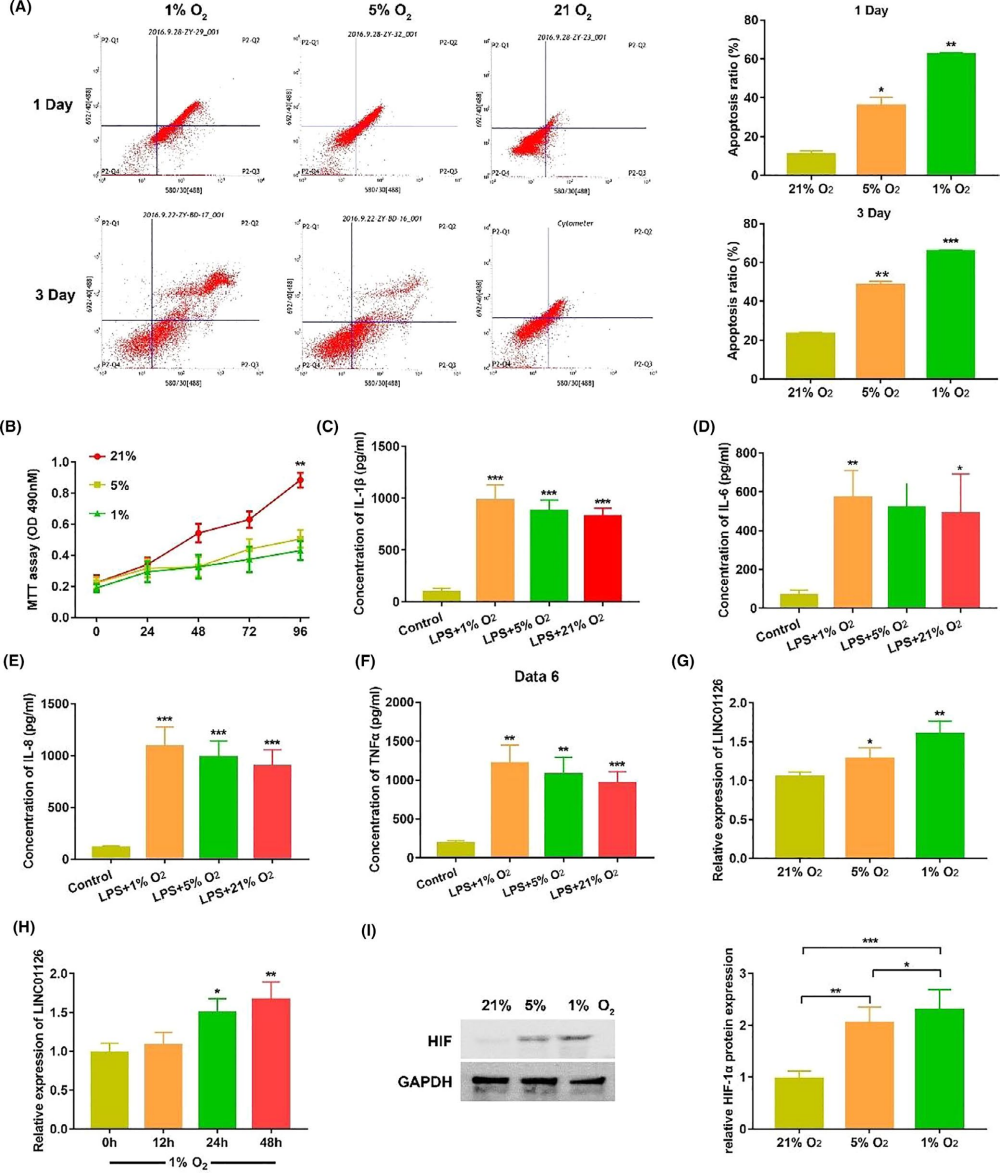
LINC01126 was upregulated in hypoxia‐induced hPDLCs. (A) Cell apoptosis ratio under hypoxia or normoxia at 1st day and 3rd day was determined by flow cytometry. (B) Cell proliferative ability at different time points under hypoxia or normoxia was determined by MTT assay. (C‐F) The secretion of inflammatory cytokines including IL‐1β IL‐6, IL‐8 and TNF‐α at different O_2_ concentrations after 24 h treatment was determined by ELISA. (G) The expression of LINC01126 at different O_2_ concentrations for 48 h was determined by qRT‐PCR. (H) The expression of LINC01126 at different time point under 1% O_2_ concentration condition by qRT‐PCR. (I)The protein expression of HIF‐1α after 24h treatment at different O_2_ concentrations by Western blot. Histogram shows quantification of the band intensities. Results are presented as mean ± SD (*p < 0.05, **p < 0.01, *** p < 0.001, compared with the control group)

hPDLCs were treated in 3 different O_2_ concentrations to imitate the hypoxia environment during the periodontitis pathogenesis. Compare to the normoxia group, hypoxia decreased the proliferative ability while increased the apoptosis rate in a concentration dependent manner (Figure 2A, B). Hypoxic condition also promoted the expression of inflammatory cytokines including IL‐1β,IL‐6, IL‐8 and TNF‐α (Figure 2C‐F). The expression level of LINC01126 was found to be significantly upregulated in 1% and 5% O_2_ group and reached peak at 48 h (Figure 2G, H). Moreover, the protein expression of HIF‐1, which is the most sensitive indicator to hypoxia was significantly upregulated under hypoxia (Figure 2I). Taken together, we disposed the cells in hypoxic conditions for 48 h in further experiment. These results indicate that hypoxia decreases the proliferation while increases apoptosis and inflammation, and LINC01126 is upregulated in hypoxia‐induced hPDLCs.

### 3.3 LINC01126 promotes hypoxia‐induced apoptosis, inflammation, suppresses proliferation in hPDLCs

**Figure 3:**
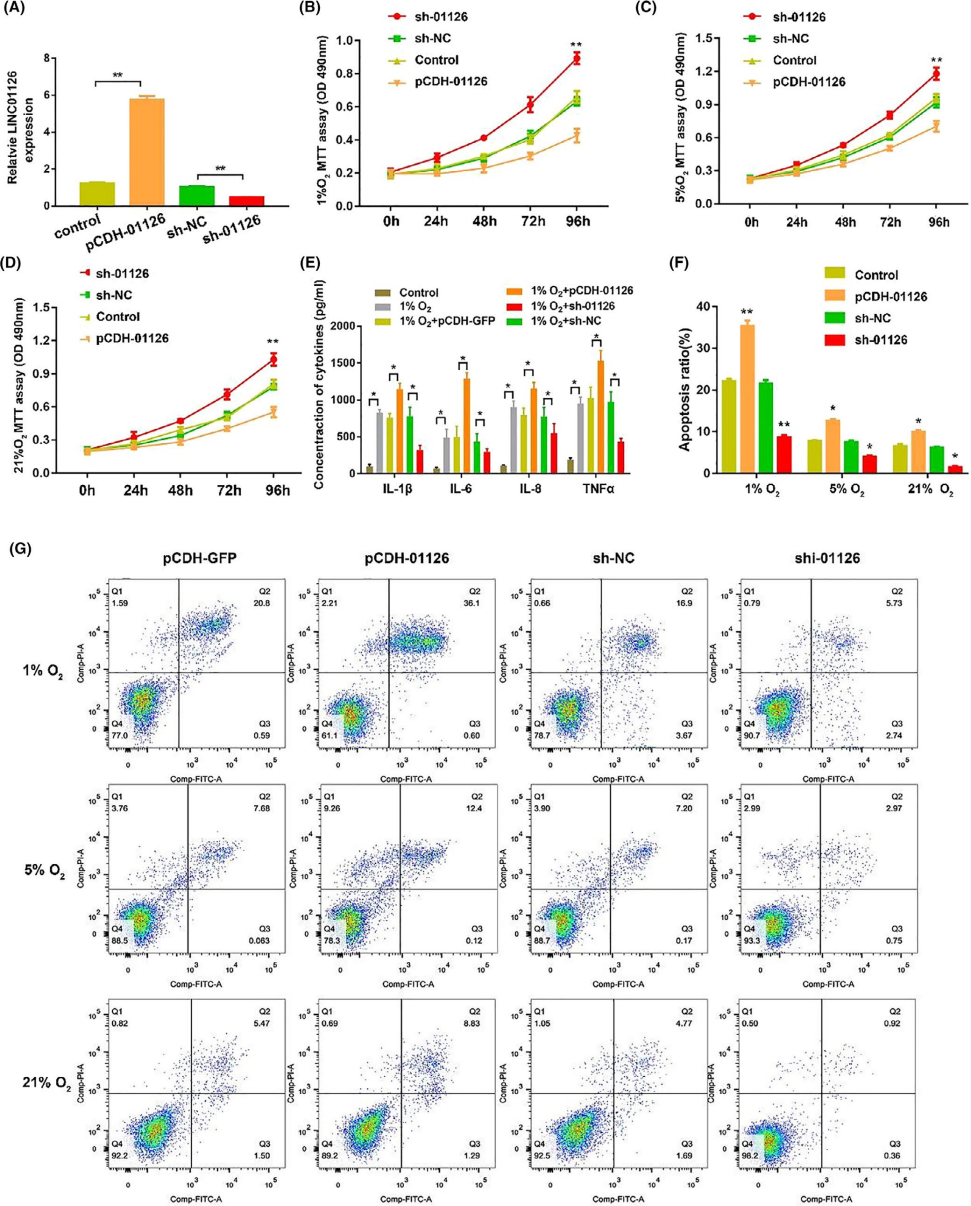
LINC01126 promoted hypoxia‐induced apoptosis, inflammation, suppressed proliferation in hPDLCs. (A) The expression of LINC01126 after transfection with pCDH‐01126, sh‐01126 and the corresponding controls. (B‐D) Cell proliferative ability after overexpression or knockdown of LINC01126 under hypoxia or normoxia. (E) ELISA showing the secretion of inflammatory cytokines including IL‐1β IL‐6, IL‐8 and TNF‐α at hypoxic environment (1% O_2_) after 24 h treatment was determined by ELISA. (F‐G) Cell apoptosis after overexpression or knockdown of LINC01126 under hypoxia or normoxia. Results are presented as mean ± SD (*P < .05, **P < .01, compared with the control group)

To determine the effect of LINC01126 in periodontitis, pCDH vector (pCDH‐01126) was transfected to overexpress LINC01126 and shRNA targeting LINC01126 (sh‐01126) was used to knock down LINC01126 expression. The transfection efficiency was more than 75% (Figure S2C,D). qRT‐PCR results were used to confirm the transfection effects in hPDLCs (Figure 3A). The proliferative capacity in each O_2_ concentration group was significantly suppressed after overexpression of LINC01126, and dramatically enhanced after knockdown of LINC01126 enhanced (Figure 3B‐D). Moreover, we found that hypoxia induced the secretion of inflammatory cytokines IL‐1β,IL‐6, IL‐8 and TNF‐α. Overexpression of linc01126 promoted the secretion of inflammatory cytokines while knockdown of LINC01126 suppressed the secretion of these inflammatory cytokines (Figure 3E). Flow cytometry analysis indicated that in each O_2_ concentration group, the number of apoptotic cells was significantly higher in LINC01126 overexpressed hPDLCs and remarkably lower in LINC01126 knockdown HPDLCs compared with the control group (Figure 3F‐G). The apoptosis ratio increased with the downregulation of the O_2_ concentration. These data suggest that overexpression of LINC01126 promotes hypoxia‐induced apoptosis, inflammation and suppresses proliferation of hPDLCs.

### 3.4 LINC01126 serves as a sponge for miR‐518a‐5p

**Figure 4:**
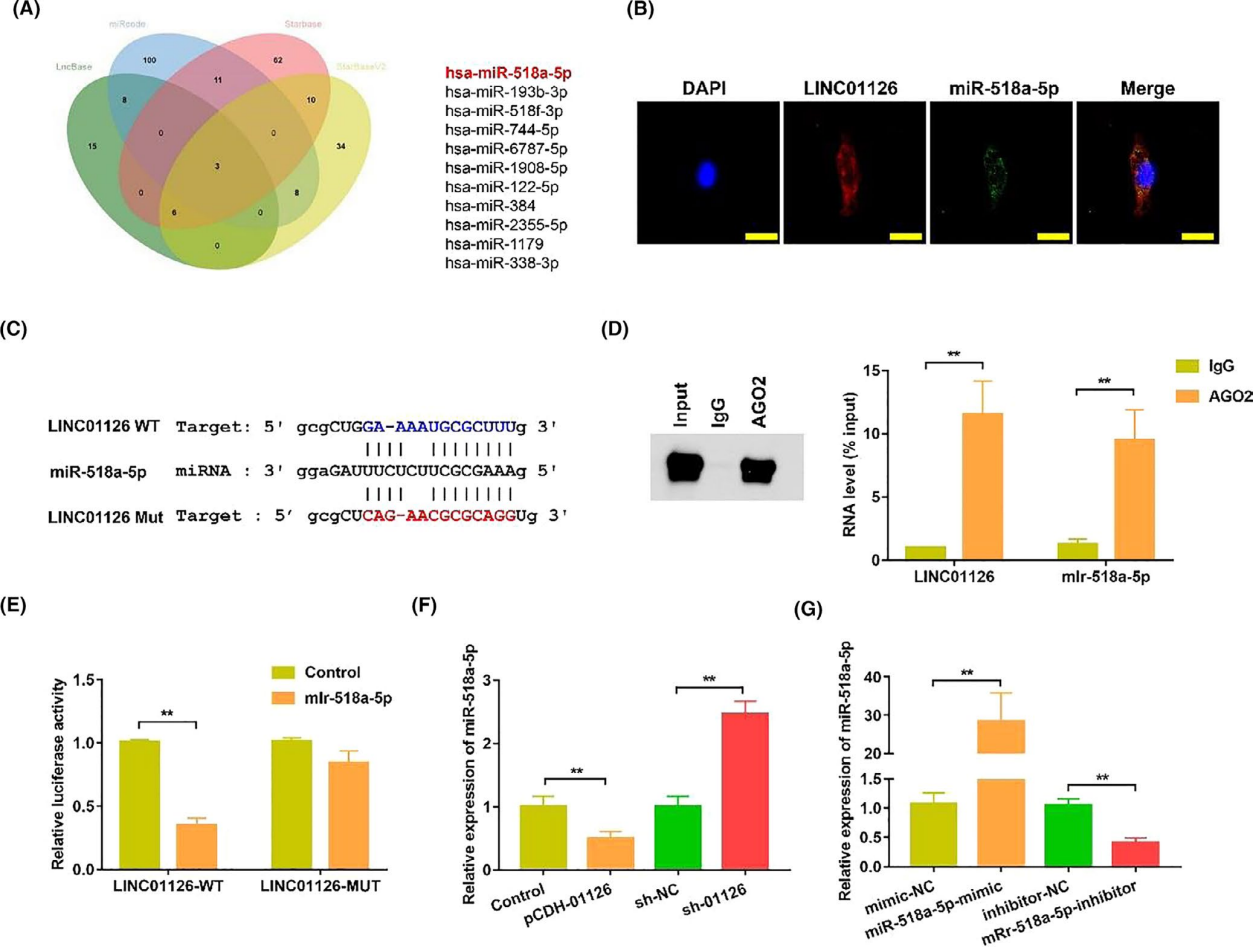
LINC01126 serves as a sponge for miR‐518. (A) Venn diagrams showing the number of potential miRNAs targeting LINC01126 (including miR‐518a‐5p). The potential miRNAs were predicted by four databases: LncBase, miRcode, Starbase, StarBaseV2. (B) Fluorescence in situ hybridization results demonstrated the subcellular location of INC01126 and miR‐518. Nuclei were stained by DAPI (blue), the scale bar = 50 μm. (C) The predicted binding of miR‐518a‐5p with LINC01126 3′‐UTR. The sequence of wild‐type (WT) and mutant (Mut) LINC01126 target sites were shown. (D) RNA immunoprecipitation assay of endogenous AGO2 binding to RNA in hPDLCs. IgG was used as the negative control. The expression of LINC01126 and miR‐518a‐5p was analysed by qRT‐PCR and the results were normalized relative to the input control. (E) Dual‐luciferase reporter assay validating the interaction between miR‐518a‐5p and LINC01126. (F) The expression of miR‐518a‐5p after transfection with pCDH‐01126, sh‐01126 and the corresponding controls. (G) The expression of miR‐518a‐5p after transfection with miR‐518a‐5p mimic, miR‐518a‐5p‐inhibitor and the corresponding controls. Results are presented as mean ± SD (**P < .01, compared with the control group)

We hypothesized that LINC01126 may act as a ceRNAsby binding to miRNAs and further competing with mRNA. We predicted that miR‐518a‐5p has a high probability of binding to LINC01126 via 4 database, including starBase (http://starbase.sysu.edu.cn/), starBaseV2 (http://starbase.sysu.edu.cn/starbase2/index.php), LncBase (http://carolina.imis.athena‐innovation.gr/diana_tools/web/index.php?r=lncbasev2%2Findex), miRcode (http://www.mircode.org/) (Figure 4A). FISH results further verified our hypothesis that both LINC01126 and miR‐518a‐5p mainly localized in cytoplasm, indicating their interaction (Figure 4B). Then, luciferase reporters containing the wide‐type (LINC01126‐WT) or mutant type (LINC01126‐MU) target site on LINC01126 were constructed to further identify whether miR‐518a‐5p could bind to LINC01126. LINC01126‐WT reporter activity was remarkably inhibited by miR‐518a‐5p in 293T while LINC01126‐MU reporter was not affected (Figure 4C, E). Moreover, RIP assay indicated that LINC01126 and miR‐518a‐5p were significantly enriched in AGO‐containing micro‐ribonucleoprotein complexes, suggesting that the AGO2 directly bound to LINC01126 and miR‐518a‐5p in hPDLCs (Figure 4D). These data indicate that miR‐518a‐5p directly binds to the LINC01126 target site. Then we compared the miR‐518a‐5p expression after LINC01126 overexpressed or knockdown in hPDLCs. The qRT‐PCR results showed that the expression of miR‐518a‐5p was significantly downregulated following LINC01126 overexpression and upregulated after LINC01126 knockdown in hPDLCs (Figure 4F). Moreover, qRT‐PCR results confirmed the transfection effects of miR‐518a‐5p mimic and miR‐518a‐5p inhibitor in hPDLCs (Figure 4G).

### 3.5 HIF‐1α is a target of LINC01126/ miR‐518a‐5p

**Figure 5:**
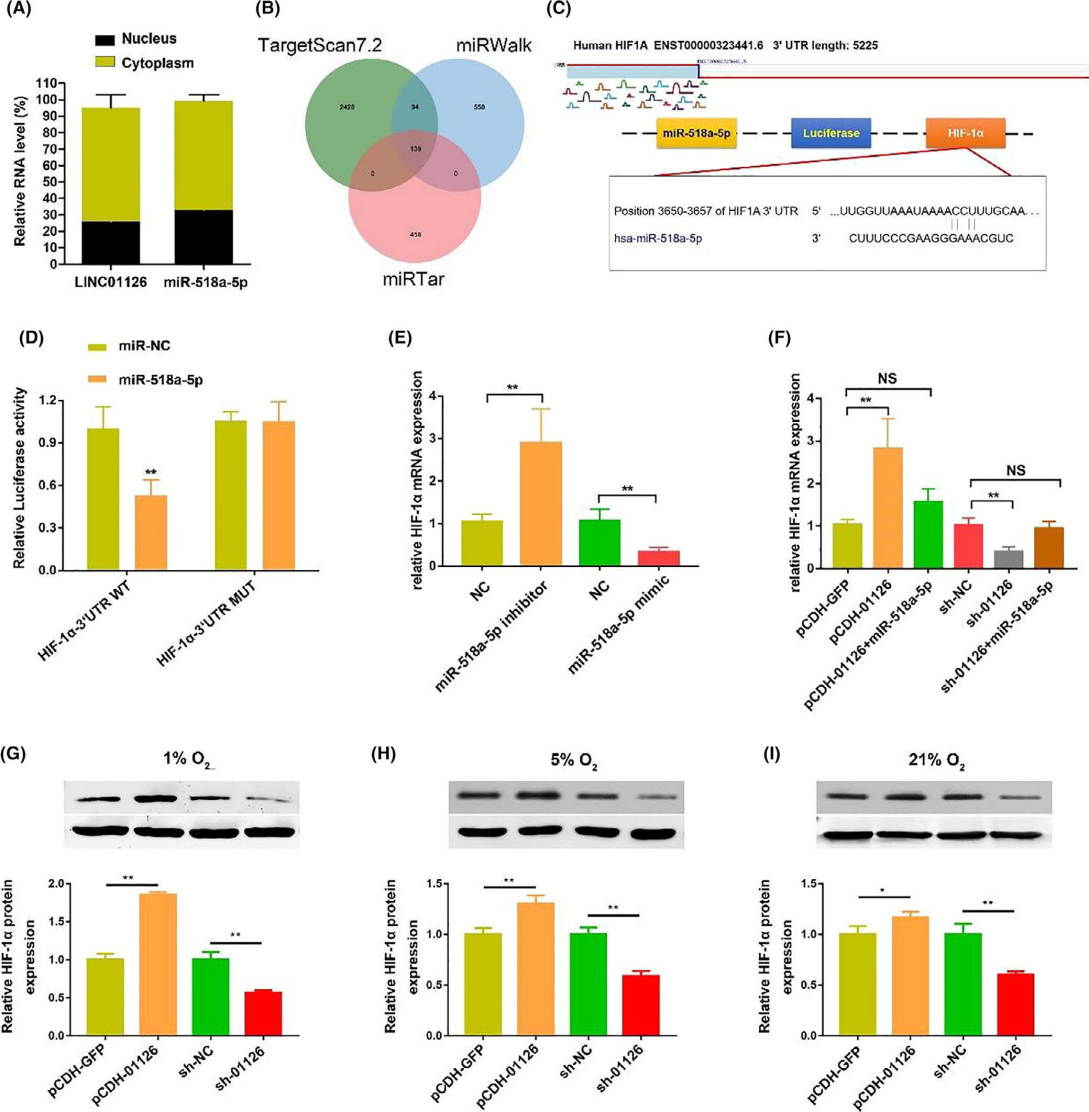
HIF‐1α is a target of LINC01126/ miR‐518a‐5p. (A) Relative expression of LINC01126 and in nuclear and cytoplasmic fractions of hPDLCs. (B) Venn diagrams showing the number of potential genes targeting miR‐518a‐5p (including HIF‐1α gene). The potential genes were predicted by three databases: TargetScan, miRWalk and miRTar. (C) The predicted binding of miR‐154‐5p with HIF‐1α 3ʹ UTR. (D) Dual‐luciferase reporter assay validating the interaction between miR‐518a‐5p and LINC01126. (E) The mRNA expression of HIF‐1α after transfection with miR‐518a‐5p mimic, miR‐518a‐5p inhibitor and the corresponding controls. (F) The mRNA expression of HIF‐1α after transfection with pCDH‐01126, LINC01126‐miR‐518a‐5p, sh‐01126, sh‐01126‐miR‐518a‐5p and the corresponding controls. (G) The protein expression level after transfection with pCDH‐01126, sh‐01126 and the corresponding controls. (H‐I) The protein expression of HIF‐1α after transfection with pCDH‐01126, sh‐01126 and the corresponding controls under different O_2_ condition. Histograms show quantification of the band intensities. Results are presented as mean ± SD (**P < .01, compared with the control group)

Cell fraction assay demonstrated the LINC01126 and miR‐518a‐5p were mainly expressed in cytoplasma in hPDLCs (Figure 5A). To further elucidate the biological mechanisms of miR‐518a‐5p in hPDLCs, we use the miRWalk (http://mirwalk.umm.uni‐heidelberg.de/), TargetScan (http://www.targetscan.org/) and miRTar (http://mirtar.mbc.nctu.edu.tw/human/ ) to predict potential targets of miR‐518a‐5p (Figure 5B). All of these three predictions showed that the 3’‐UTR of hypoxia‑inducible factor‑1α (HIF‑1α) contained a miR‐518a‐5p binding site (Figure 5C). HIF‐1α is one of the most sensitive and important molecule in response to Hypoxic conditions in hPDLCs. Therefore, we identified HIF‐1α as a putative miR‐518a‐5p target. Then, HIF‐1α 3ʹ UTR and its mutant containing the putative miR‐518a‐5p binding sites were cloned into the downstream luciferase reporter. Compared with the control group, luciferase reporter activity was significantly reduced in miR‐518a‐5p transfected hPDLCs, and this reduction was relieved in mutated HIF‐1α 3’UTR (Figure 5D). Moreover, after transfection of miR‐518a‐5p mimics, the expression of HIF‐1α decreased, while the level of HIF‐1α increased after transfection of miR‐518a‐5p inhibitors in hPDLCs (Figure 5E). Additionally, the expression of HIF‐1α was significantly upregulated in cells overexpressed LINC01126, whereas co‐transfection with pCDH‐01126 and miR‐518a‐5p inhibitor significantly reversed HIF‐1α expression levels. Downregulation of LINC01126 significantly downregulated the expression of HIF‐1α,while transfection with sh‐01126 and miR‐518a‐5p mimic synchronous had a reversed effect (Figure 5F). Under hypoxia or normoxia, the protein expression of HIF‐1α was significantly decreased following downregulation of LINC01126. On the contrary, the HIF‐1α expression level was significantly increased after overexpression of LINC01126 (Figure 5G‐I). Altogether, these data indicate that HIF‐1α acts as a downstream target of LINC01126/ miR‐518a‐5p axis.

### 3.6 HIF‐1α promotes hypoxia‐induced apoptosis and suppressed proliferation in hPDLCs

**Figure 6:**
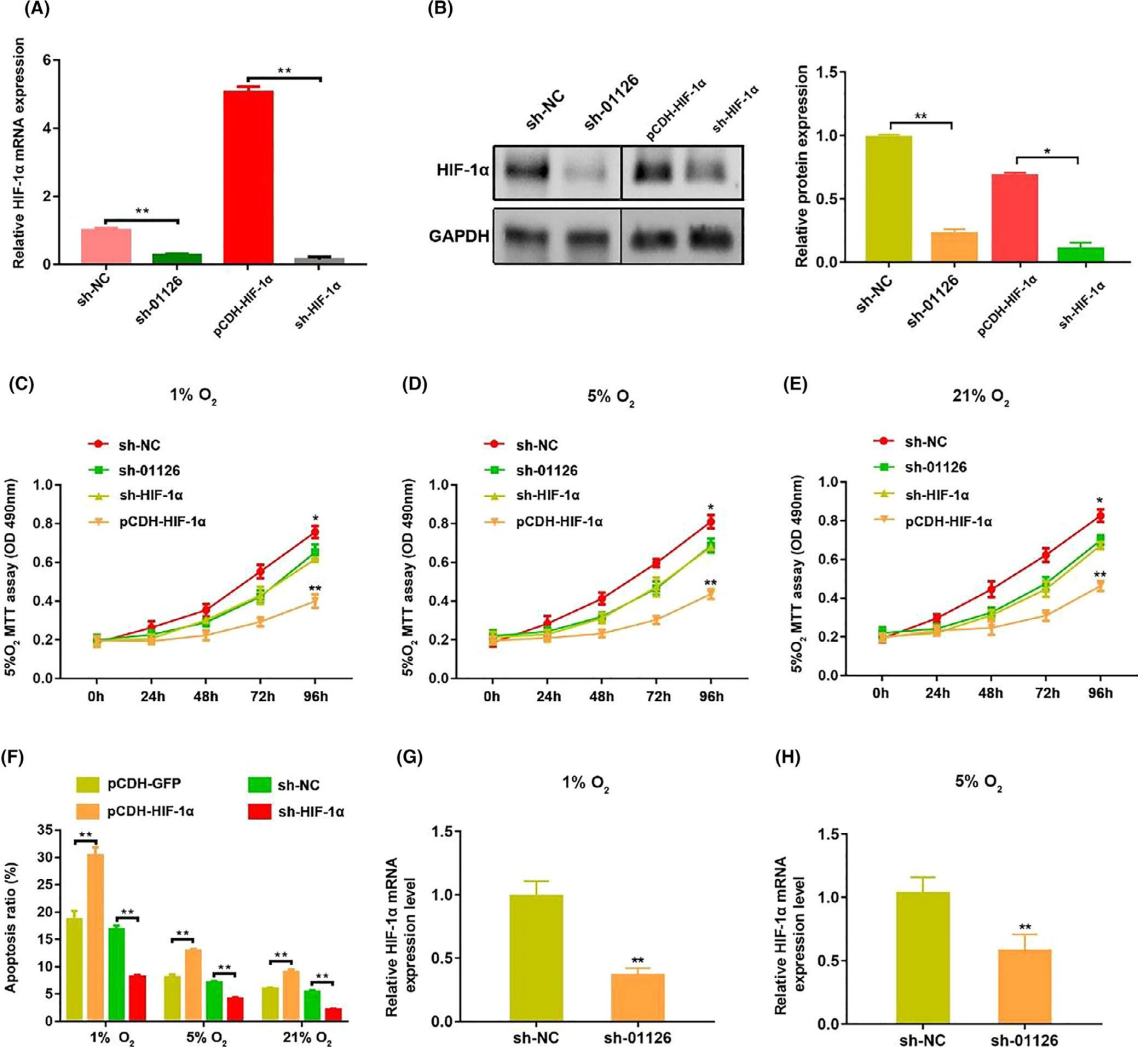
HIF‐1α promotes hypoxia‐induced apoptosis and suppressed proliferation in hPDLCs. (A, B) The mRNA and protein expression of HIF‐1α after transfection with transfection with sh‐LINC01126, pCDH‐HIF‐1α, sh‐HIF‐1α and the corresponding controls. Histogram shows quantification of the band intensities. (C‐E) Cell proliferative ability after overexpression or knockdown of HIF‐1αunder different O_2_ concentrations was determined by MTT assay. (F) Cell apoptosis ratio after overexpression or knockdown of HIF‐1α under different O_2_ concentrations. (G‐H) The expression of HIF‐1α mRNA after knockdown of LINC01126 under hypoxia (1% O_2_, 5% O_2_). Results are presented as mean ± SD (*P < .05, **P < .01, compared with the control group)

To explore the function of HIF‐1α under hypoxic conditions in hPDLCs, we first detect the transfection efficiency of sh‐HIF‐1α, pCDH‐HIF‐1α and the corresponding controls. qRT‐PCR and Western blot results demonstrated that HIF‐1α was successful overexpressed or knocked down in hPDLCs (Figure 6A, B). Moreover, we found that knockdown of HIF‐1α significantly inhibited proliferation capability of hPDLCs under hypoxic conditions, while overexpression of HIF‐1α promoted the proliferation capability (Figure 6C‐E). The apoptosis ratio remarkably decreased when HIF‐1α were knockdown and significantly increased after overexpression of HIF‐1α no matter under hypoxia or normoxia (Figure 6F). In addition, depletion of HIF‐1α reduced the expression of LINC01126 under hypoxia (Figure 6G, H), indicating that LINC01126 is hypoxia‐inducible and it can be regulated by HIF‐1α in turn.

### 3.7 LINC01126/miR‐518a‐5p/HIF‐1α aggravates periodontitis pathogenesis via activating MAPK pathway

**Figure 7:**
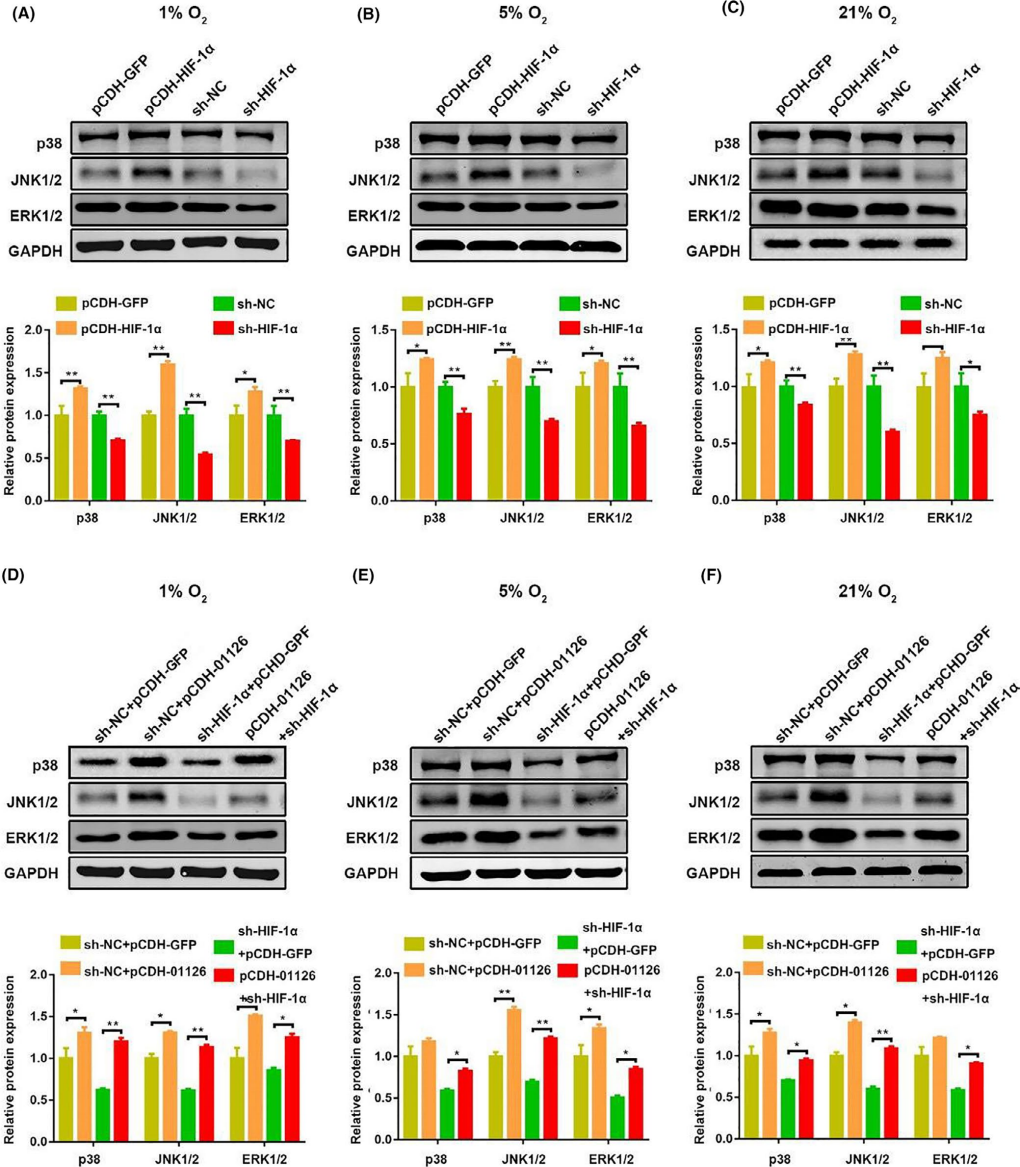
LINC01126/miR‐518/HIF‐1α aggravates periodontitis pathogenesis via activating MAPK pathway. (A‐C) The protein expression of p38, JNK1/2, ERK1/2 after transfection with pCDH‐HIF‐1α, sh‐HIF‐1α and the corresponding controls under different O_2_ concentrations. Histogram shows quantification of the band intensities. (D‐F) The protein expression of p38, JNK1/2, ERK1/2 after transfection with pCDH‐01126, sh‐HIF‐1α/ pCDH‐01126 and the corresponding controls under different O_2_ concentrations. Histogram shows quantification of the band intensities. Results are presented as mean ± SD (*P < .05, **P < .01, compared with the control group)

Mitogen‐activated protein kinases (MAPKs), including extracellular signal‐regulated kinase (ERK), c‐Jun N‐terminal kinase (JNK) and p38‐MAPK, are an important pathway in cell proliferation and inflammation ^28^. The GSEA analysis predicted that MAPK pathway was associated with a majority of differentially expressed genes including LINC01126 in periodontitis. To investigate whether LINC01126/HIF‐1α aggravates periodontitis development via accelerated MAPK pathway, we first use the Western blot to measure the protein expression of the total p38, ERK1/2, and JNK in sh‐HIF‐1α, pCDH‐01126/HIF‐1α‐treated hPDLCs. The results indicated that MAPK pathway was markedly decreased in HIF‐1α knockdown group, while increased in the HIF‐1α overexpressed group (Figure 7A‐C). Moreover, we co‐transfected PDCLs with pCDH‐01126 and sh‐HIF‐1α. The results showed that overexpression of lnc01126 activated the MAPK pathway. However, co‐transfection with pCDH‐01126 and sh‐HIF‐1α alleviated the activation of MAPK pathway (Figure 7D‐F).

## 4 DISCUSSION

Accumulating evidence has revealed that numerous lncRNAs have regulatory roles in a variety of cellular process ^17^, ^29^, ^30^. However, there is lack of lncRNAs associated with periodontitis. Only one study demonstrated that lncRNA HIF1A‐AS1 and HIF1A‐AS2 regulating osteogenic differentiation via regulating HIF‐1α in hPDLCs under hypoxia ^20^. In present study, to explore the expression pattern of lncRNAs in periodontitis‐induced hypoxia, we innovatively investigate lncRNA signatures in 3 pair of gingival tissues from periodontitis patients and healthy controls via a lncRNA microarray. We focus on the aberrantly expressed lncRNAs and chose the most upregulated LINC01126 to further verify its function in periodontitis‐induced hypoxia. We identified that LINC01126 was highly expressed in gingival tissues from patients with periodontitis. In vitro experiments demonstrated that hypoxia induced decrease of LINC01126 expression.

Maintaining active proliferation ability and anti‐inflammation and anti‐apoptosis role of hPDLSCs is important for the periodontium to resist periodontitis‐induced hypoxia ^31^, ^32^, ^33^. Overexpression of LINC01126 promoted apoptosis, inflammation, suppressed proliferation whereas knockdown decreased the apoptosis, inflammation, enhanced proliferation of hPDLCs under hypoxia. These data suggest LINC01126 may become a new target to treat periodontitis. miRNAs belonging to non‐coding RNAs family can bind to the complementary sequences in 3’ UTRs of the target mRNA and further negatively regulate the target gene expression through inhibiting translation or destabilizing the target mRNA. lncRNAs can competely bind to miRNA and further affect the targets which bind to miRNAs ^21^. Dysregulated miRNAs are reported in periodontitis pathogenesis like microRNAs let‐7a, miR‐125b, miR‐100 and miR‐21 ^34^. They play important roles in regulating a variety of biological processes including cellular proliferation, differentiation, apoptosis as well as inflammatory processes ^35^. In present study, we found that LINC01126 is in the cytoplasm of hPDLCs and we hypothesize that LINC01126 may function as a sponge for miRNAs. Bioinformatics algorithms predicted that LINC01126 contains a binding site of miR‐518a‐5p. miR‐518a‐5p has been reported to be sponged by several non‐coding RNAs ^36^, ^37^. For example, circ_0074027 could sponge miR‐518a‐5p to release its suppression on IL17RD and circ_0078607 could sponge oncogenic miR‐518a‐5p to suppressed ovarian cancer progression ^36^, ^37^. MiR‐518a‐5p is also reported to be negatively regulated by Cysteine‐rich 61 which is an important proinflammatory cytokine via the MAPK cascade, indicating miR‐518a‐5p is involved in inflammation regulation ^38^. In this study, we found that LINC01126 negatively regulated miR‐518a‐5p expression by acting as a sponge. Luciferase reporter assays and RIP assay further confirmed that LINC01126 and miR‐518a‐5p are directly interacted with each other in hPDLCs.

Hypoxia‐inducible factor‐1 (HIF‐1) is composed of HIF‐α (HIF‐1α, HIF‐2α and HIF‐3α) and HIF‐β subunits and HIF‐1α is the dominant functional subunit. Under hypoxic conditions, the expression of HIF‐1α is highly increased while under normoxia, it is unstable and easily degraded ^39^, ^40^. As a transcription factor, HIF‑1α regulates the downstream genes in response to hypoxia ^41^. As periodontal pockets are characterized by a reduced oxygen level, HIF‐1α also plays an important role in periodontitis‐induced hypoxic environment ^33^, ^41^. It has been reported to be the target of several miRNAs including miR31HG, miR‐143‐5p and miR‐217 ^42^, ^43^, ^44^. However, the regulatory mechanism of HIF‑1α mRNA remains unclear. In our study, we identified HIF‐1α as a direct target of miR‐518a‐5p via miRWalk, TargetScan and miRTar combined analyses. LINC01126/miR‐518a‐5p regulated hPDLCs proliferation, apoptosis and inflammation under hypoxic conditions via targeting HIF‐1α. The putative binding of miR‐518a‐5p to HIF‐1α was confirmed by luciferase reporter assays. The expression of HIF‐1α was negatively correlated with miR‐518a‐5p and positively regulated by LINC01126. Co‐transfection with miR‐518a‐5p mimic and sh‐01126 counteracting the downregulation effects of sh‐HIF‐1α to HIF‐1α. Constantly, co‐transfection with pCDH‐01126 and miR‐518a‐5p mimic reversed the upregulation effect of pCDH‐01126 to HIF‐1α. These results indicate that HIF‐1α acts as a downstream target of LINC01126/ miR‐518a‐5p axis. Silencing HIF‐1α promoted cell proliferation, inhibited apoptosis while overexpression of HIF‐1α significantly promoted proliferation, suppressed apoptosis.

As a proliferative and antiapoptotic signalling pathway, ERK/p38/JNK MAPK signalling pathways are involved in varieties of cellular activities, including proliferation, differentiation and survival ^28^, ^45^. Previous study demonstrated that MAPK signalling pathway promotes HIF‐1α expression but does not affect the mRNA expression ^46^. However, whether HIF‐1α affects MAPK pathway remain controversial. In this study, GSEA plot predicted that the majority of differentially expressed genes were involved in the MAPK signalling pathway, so we further verified our prediction. We observed that no matter in hypoxia or normoxia conditions, knockdown of HIF‐1α downregulated the protein expression of p38, ERK, JNK, while overexpression of HIF‐1α enhanced the MAPK signalling pathway. Moreover, evidence showed that overexpression of LINC01126 activated the MAPK pathway, but co‐transfection with pCDH‐01126 and sh‐HIF‐1α alleviated the activation of MAPK pathway in hPDLCs. Taken together, these results suggest that the LINC01126/miR‐518a‐5p/ HIF‐1α‐related periodontitis pathogenesis of hPDLCs is modulated through a MAPK signalling pathway.

The limitations of our study are that the downstream targets of LINC01126 may not limit to the miR‐518a‐5p/HIF‐1α axis, and further studies need to be performed to explore other downstream targets of LINC01126 to better understand the role of LINC01126 in periodontitis pathogenesis. Moreover, in vivo study should be carried out in further experiments to validate our findings.

**Figure 8:**
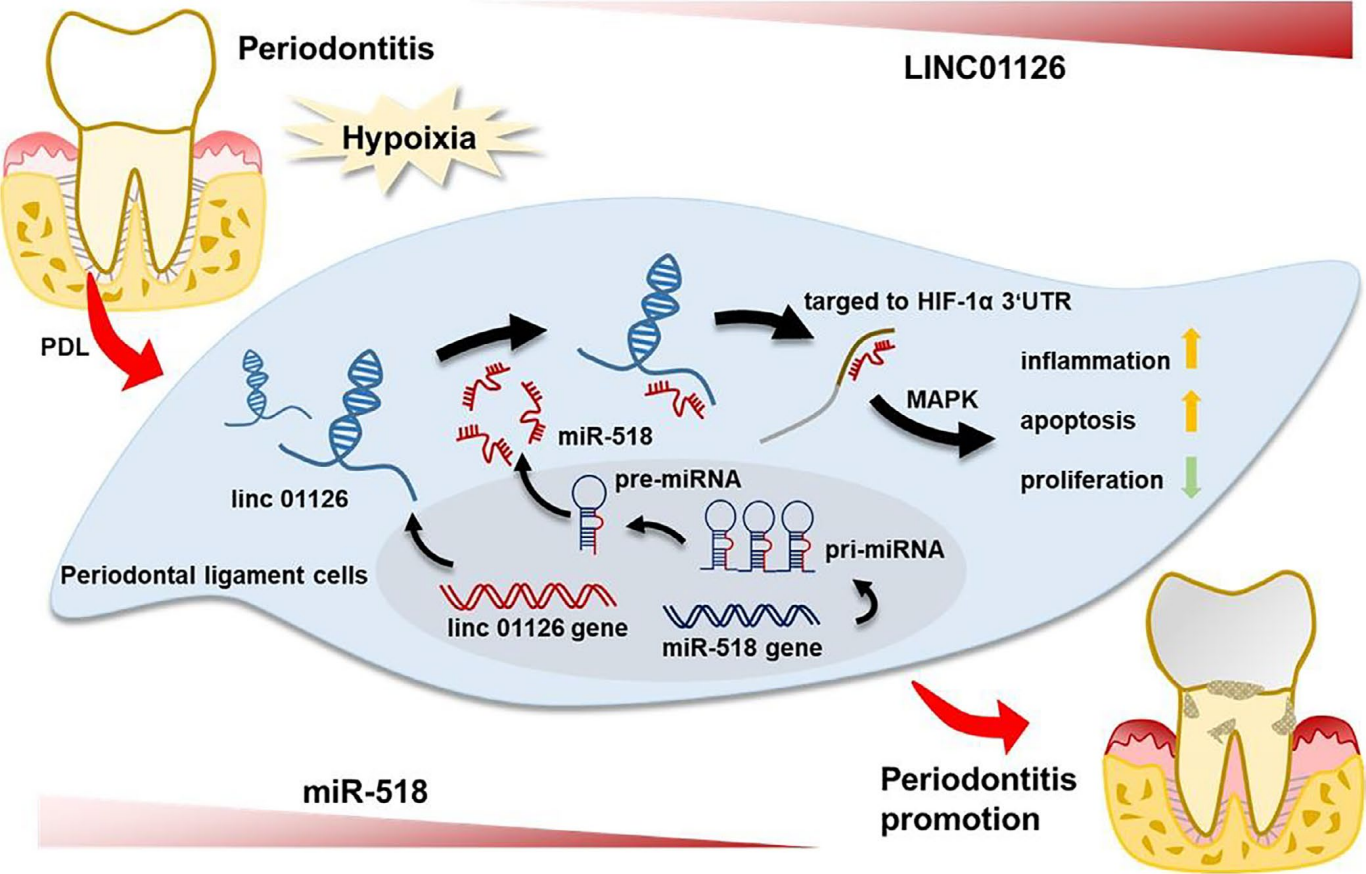
Schematic model showing the regulation periodontitis pathogenesis by of LINC01126

In conclusion, our results suggest that LINC01126 positively promotes apoptosis, inflammation while negatively regulates proliferation of hPDLCs under hypoxia. Mechanistically, LINC01126 inhibits miR‐518a‐5p expression, which directly regulates HIF‐1α, and further activates the MAPK pathway to regulate the hPDLCs under hypoxia (Figure 8). This lnc01126/miR518/HIF‐1α axis may providing possible clues for LINC01126‐based periodontal therapeutic approaches.

## CONFLICT OF INTEREST

The authors declare no conflict of interest.

## AUTHOR CONTRIBUTIONS

XNZ and MZ designed the study. MY and HH collected the data. HMZ, YZ and JL analysed and interpreted the data. XNZ and YNH wrote the original draft. ST and YY involved in critical reviewing of the manuscript. XNZ, YNH, YY and JL performed bioinformatics analysis.

## Supporting information

Fig S1‐S2

Table S1‐S3

## Data Availability

The data that support the findings of this study are available from the corresponding author upon reasonable request.
