# Supplementary material for: Long non‐coding RNA 01126 promotes periodontitis pathogenesis of human periodontal ligament cells via miR‐518a‐5p/HIF‐1α/MAPK pathway
Source: Cell Prolif. 2020 Nov 24;54(1):e12957. doi: 10.1111/cpr.12957 (PMC7791173; doi:10.1111/cpr.12957)
Supplement: Supplementary file 1 — Fig S1‐S2 [file CPR-54-e12957-s001.docx]

**Supplementary Figure legends**

**
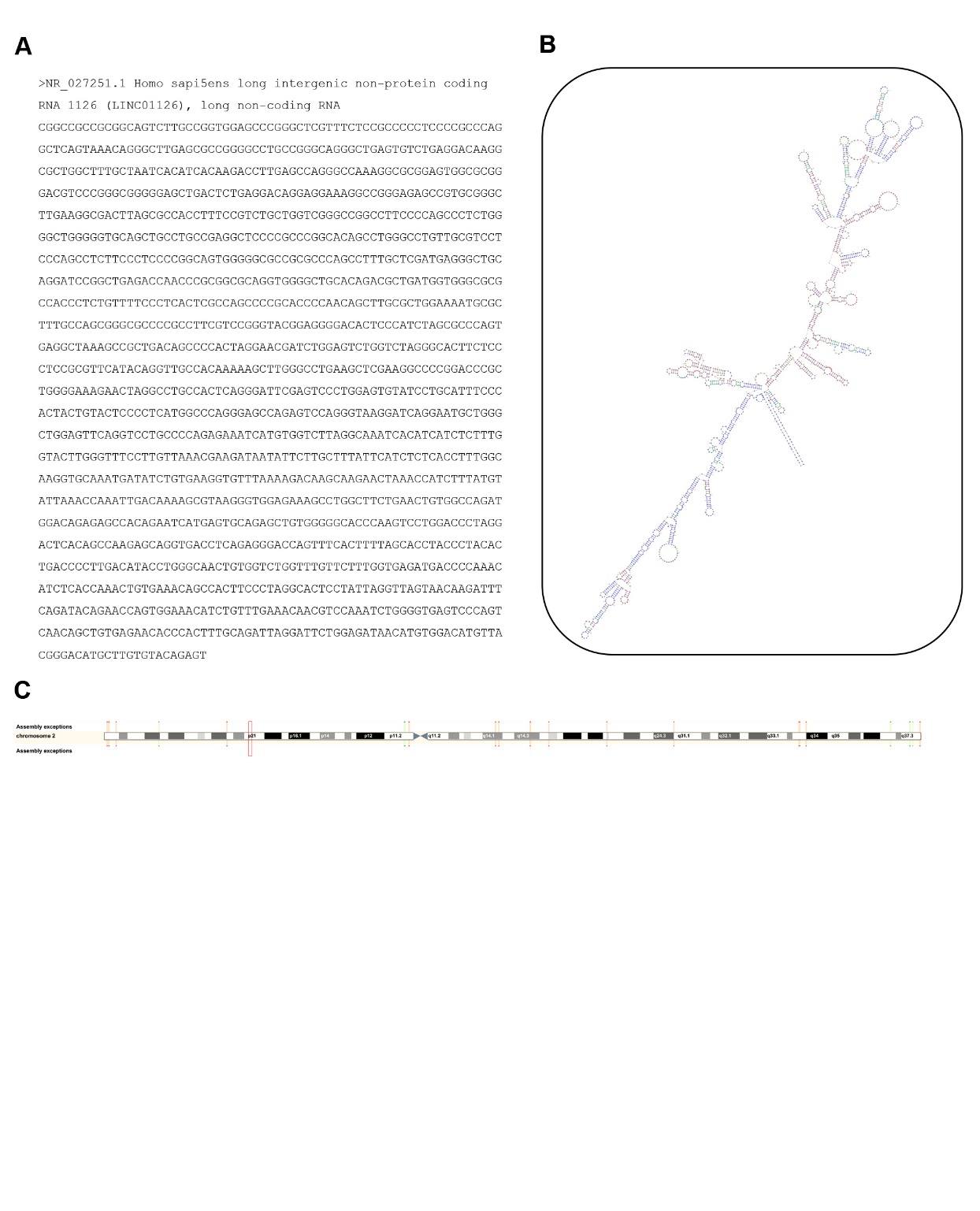
**

**Supplement Figure 1.** (A) The full length sequence of LINC01126 from the GeneCards website (https://www.genecards.org/). (B) Secondary structure of LINC011126 from GeneCards website. (C) LINC01126 Gene in genomic location (2p21) according to GeneLoc; bands according to Ensembl.

**
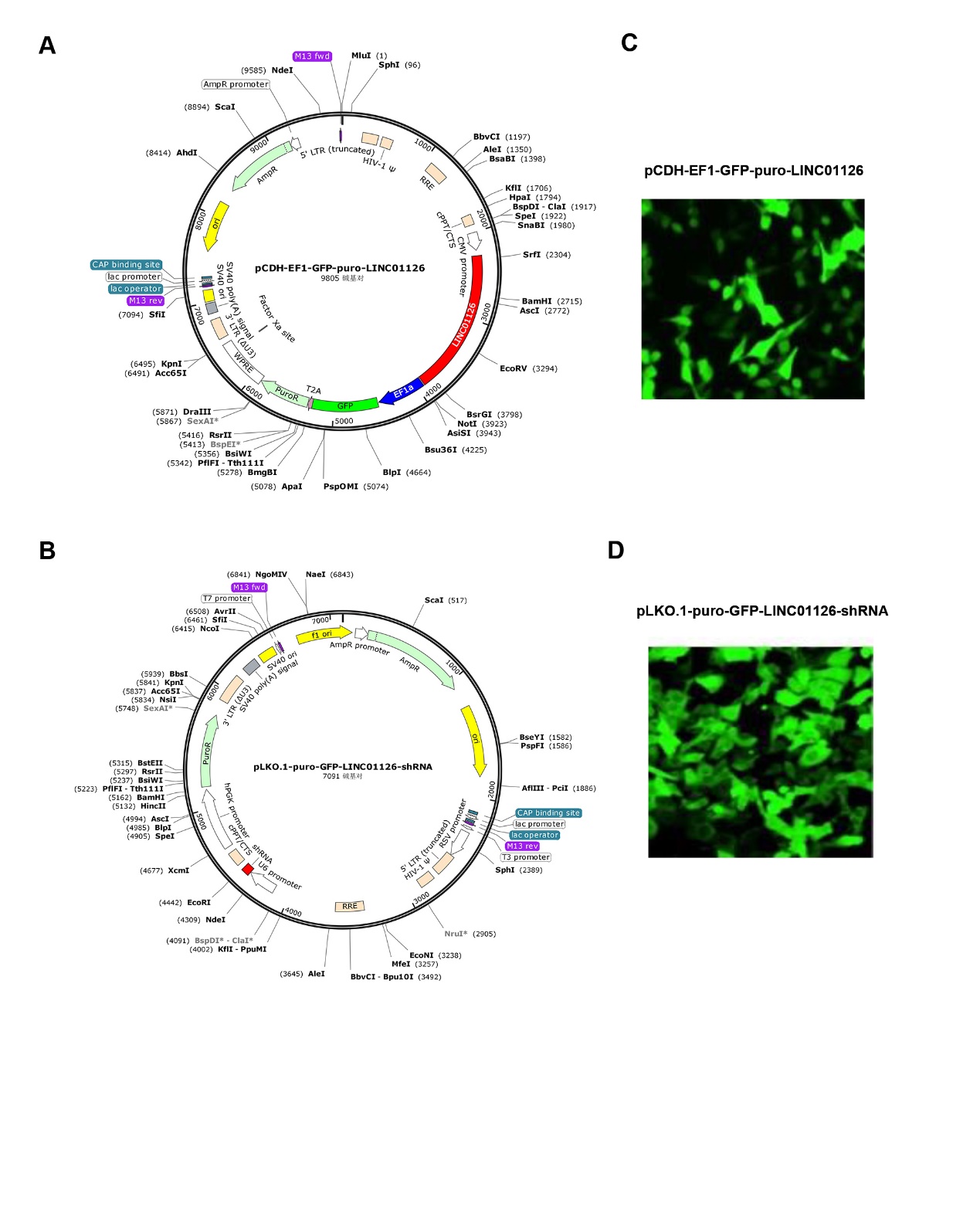
**

**Supplement Figure 2.** (A, B) Schematic diagram illustrating the pCDH-EF1-GFP-puro-LINC01126 vector and pLKO.1-puro-GFP-LINC01126-shRNA vector. (C, D) Transfection of green fluorescent protein (GFP) in hPDLSC.
