# Supplementary material for: Long non‐coding RNA 01126 promotes periodontitis pathogenesis of human periodontal ligament cells via miR‐518a‐5p/HIF‐1α/MAPK pathway
Source: Cell Prolif. 2020 Nov 24;54(1):e12957. doi: 10.1111/cpr.12957 (PMC7791173; doi:10.1111/cpr.12957)
Supplement: Supplementary file 2 — Table S1‐S3 [file CPR-54-e12957-s002.docx]

**Supplementary Materials**

**Supplement Table 1. The sequences of the RNA oligoribonucleotides.**

| miRNAs |  |
| --- | --- |
| miR-518 mimic | CUGCAAAGGGAAGCCCUUUC |
| miR-518 inhibitor | GAAAGGGCUUCCCUUUGCAG |
| mimic-NC | UCACAACCUCCUAGAAAGAGUAGA |
| inhibitor-NC | UCUACUCUUUCUAGGAGGUUGUGA |

**Supplement Table 2. The sequences of shRNA**

| sh-RNA |  |
| --- | --- |
| sh-01126 | GGTCTTAGGCAAATCACATCA |
| sh-HIF-1α | GAGUGUAACAUCGUAGUAA |
| sh-NC | TTCTCCGAACGTGTCACGTTTC |

**Supplement Table 3. Nucleotide sequence of primers used in qRT-PCR.**

| Gene | Forward primer | Reverse primer |
| --- | --- | --- |
| LINC01126 | TGAGCCACAACCAACAGCAG | GTCAAGAGGAGAGCCAGAGATG |
| lnc KLLP | ATAGGATCCAGAAGAAATAGCAAGTGCCGAGAA | ATAGCGGCCGCCTTTACGTTCTGGGATACATGT |
| LINC01314 | GACTAAGTCCTTTATCCCTCCCC | GGACTATCCATGAACACAAAGAGGG |
| LINC545726 | GCTCAGGTGCTTTCACTAATGTCTC | CGCGGATCCCTTTACGTTCTGGGATACATGTGCAG |
| HIF-1α | GAACGTCGAAAAGAAAAGTCTCG | CCTTATCAAGATGCGACTCACA |
| miR-518a-5p | CTGCAAAGGGAAGCCCTT | TATCCAGTGCGTGTCGTG |
| β-actin | GGTCACCAGGGCTGCTTTTA | GGATCTCGCTCCTGGAAGATG |
